# Weight gain in patients with severe atopic dermatitis treated with dupilumab: a cohort study

**DOI:** 10.1186/s12895-020-00103-0

**Published:** 2020-09-22

**Authors:** Emma Kristin Johansson, Lina Ulrika Ivert, Baltzar Bradley, Maria Lundqvist, Maria Bradley

**Affiliations:** 1 Dermatology and Venereology Unit, Department of Medicine Solna, Karolinska Institutet, SE-171 77 Stockholm, Sweden; 2 Dermatological and Venereal Clinic, Södersjukhuset, SE-118 83 Stockholm, Sweden; 3 Department of Dermatology, Karolinska University Hospital Solna, SE-171 76 Stockholm, Sweden

**Keywords:** Adverse event, Atopic eczema, Biologics, Side effect

## Abstract

**Background:**

Dupilumab, targeting the interleukin-4α receptor and inhibiting the action of interleukin-4 and interleukin-13, was recently approved for treatment of moderate to severe atopic dermatitis. There is limited data on long-term effects and safety among patients with severe atopic dermatitis treated with dupilumab. Weight gain was observed among patients treated with dupilumab in our clinic. The aim was to describe weight change in a cohort study of patients with severe atopic dermatitis treated with dupilumab from baseline to follow-up after 12 months, and to analyze if weight change was associated with effect of treatment, reported appetite, and/or disturbed night sleep due to itching.

**Methods:**

All patients with atopic dermatitis receiving systemic treatment at the Unit of Dermatology, Karolinska University Hospital, have been registered and monitored consecutively since January 2017. This cohort constituted all patients who started treatment on dupilumab or methotrexate between 10 January 2017 and 30 June 2019 with at least 6 months of follow-up within the study period. The following variables were monitored at start of and during treatment: Eczema Severity Score Index, Patient-Oriented Eczema Measure, visual analogue scale for pruritus 10 cm, Montgomery-Åsberg Depression Rating Scale, Dermatology Life Quality Index, and weight. Data analyses were performed using two-sample Wilcoxon-Mann-Whitney rank-sum test, or the Wilcoxon matched-pairs sign-rank test with a *p*-value < 0.05 considered as statistically significant.

**Results:**

Patients treated with dupilumab (*n* = 12) gained weight (mean 6.1 kg, range [0.1–18.0], *p* = 0.002) after 1 year on treatment. The majority of patients showed a good response to treatment with dupilumab (*n* = 11); at follow-up at 6, 9, or 12 months, they reached EASI-90 (*n* = 6), EASI-75 (*n* = 4), or EASI-50 (*n* = 1). There was no significant association between weight gain and treatment response, reported appetite, or disturbed night-sleep due to itch. Patients treated with methotrexate showed no significant weight change (*n* = 8).

**Conclusions:**

To our knowledge, this is the first report on a possible association between weight gain and dupilumab treatment; the extent of the association is yet to be seen, as is the mechanism behind this finding.

## Background

Atopic dermatitis (AD) (i.e., atopic eczema) is a common inflammatory skin disease characterized by dry skin and episodes of itchy skin lesions [1]. Topical therapy with emollients and anti-inflammatory glucocorticoids and/or calcineurin inhibitors constitutes the first-line treatment [2]. Systemic immunosuppressive treatment with ciclosporin, methotrexate, azathioprine or mycophenolic acid is commonly used for refractory cases with severe AD [3]. Biologics targeting the interleukin (IL)-4α receptor (dupilumab) have recently been approved for moderate to severe AD [4]. There is limited data on long-term effects and safety among patients with severe AD treated with dupilumab. At our clinic at the Unit of Dermatology, Karolinska University Hospital, several patients receiving treatment with dupilumab reported weight gain.

To our knowledge, there is no previously published report that weight gain could be associated with dupilumab treatment among patients with AD. However, patients with psoriasis, rheumatoid arthritis (RA) or inflammatory bowel disease (IBD) treated with other biologics are reported to put on weight during treatment [5–8]. The mechanism behind the weight gain is not fully understood. One possible explanation could be that the disease leads to energy consumption, meaning that the patient will gain weight if treatment is successful and diet is unchanged. Patients with AD scratch due to itching during night sleep and suffer from sleep disturbance more frequently than healthy controls [9, 10]. Jenney et al. have previously described that scratching during sleep increased oxygen consumption in AD patients [11], and others have reported that children and adolescents with severe AD and disturbed night sleep had a reversible short stature [12]. Both these findings could be due to increased energy expenditure; weight gain during treatment with dupilumab might be a consequence of a reversal in increased energy expenditure.

Another possible explanation is that inhibition of the IL-4α receptor interferes with signaling pathways in other organs or systems in the body. The IL-4α receptor signaling is a crucial regulator in the development of post-natal brown fat [13]. Brown fat is associated with cold-induced non-shivering thermogenesis and maintained activation of brown fat is negatively correlated with obesity [14]. Studies on mice have shown that overexpression of IL-13 prevents high-fat diet-induced weight gain without affecting food consumption [15], and administration of IL-4 increased brown fat mass and the thermogenic capacity to decrease established obesity [16]. As IL-4 and IL-13 have an anti-inflammatory effect in the fat metabolism, and treatment with dupilumab blocks IL-4 and IL-13, this might interfere with activation of brown fat and thus increase the risk for obesity in patients with AD.

In this cohort study, we aimed to describe weight change among patients with severe AD treated with dupilumab from baseline to follow-up after 12 months. Further, we wanted to analyze if weight change was associated with effect of treatment, reported appetite, and/or disturbed night sleep due to itching.

## Methods

We used prospectively collected data from our research register of patients with severe AD who had received systemic immunosuppressive treatment. All patients with AD on systemic treatment are consecutively registered since 10 January 2017. Data on sex, age, atopic comorbidity, filaggrin mutations, IgE sensitization, and contact allergy, among other things, are recorded at inclusion [17] and the following variables are monitored at start of and during treatment: Eczema Severity Score Index (EASI) [18], Patient-Oriented Eczema Measure (POEM) [18], visual analogue scale for pruritus 10 cm (VAS) [19], Montgomery-Åsberg Depression Rating Scale (MADRS) [20], Dermatology Life Quality Index (DLQI) [21], and weight. Questionnaire data used in this study, was collected as standard practice from all patients with AD at each visit to the dermatologist (at start of treatment, after 1 month on treatment, after 3 months and every third to sixth month thereafter). In total, 41 patients started treatment with dupilumab and/or methotrexate between 10 January 2017 and 30 June 2019. Patients with follow-up of less than 6 months (*n* = 15) or missing values regarding weight (*n* = 8) were excluded. The final study population consisted patients with AD who had undergone treatment with dupilumab (*n* = 12) and/or methotrexate (*n* = 8). All patients who started treatment with dupilumab had previously been treated with methotrexate, but only two of them were treated with both methotrexate and dupilumab and had a follow-up of at least 6 months within the study period. These two patients are included in both the methotrexate group and the dupilumab group in the analyses.

### Definitions

Successful treatment was defined as 75% improvement in EASI score (EASI-75) from baseline to follow-up at 6, 9, or 12 months and/or improvement in EASI score ≥ 6.6 (minimal clinically important difference) from baseline to follow-up at 6, 9, or 12 months [22]. Minimal clinically important difference in symptoms was defined as a reduction ≥4 in POEM score [22].

Reduced appetite was asked about in MADRS: “*Do you experience a reduced appetite compared with when feeling well? Scale 0–6; 0: Normal or increased appetite; 2: Slightly reduced appetite; 4: No appetite / food is tasteless 6: Need persuasion to eat.”*

Disturbed night sleep due to itching was asked about in POEM: *“Over the last week have many nights has your sleep been disturbed because of the eczema? Scale 0-6; 0 No days 1: 1–2 days 2: 3–4 days 3: 5–6 days 4: Every day”.*

### Statistics

Background characteristics and outcome measures at baseline were expressed as percentage of the total number of individuals observed, or mean value, and 95% confidence intervals (95% CI). *P* values were calculated with the two-sample Wilcoxon-Mann-Whitney rank-sum test, and *p* < 0.05 was considered significant. The Wilcoxon matched-pairs sign-rank test was used to compare outcome measures (weight, EASI, POEM, VAS, DLQI, MADRS) at baseline with outcome measures at follow-up among patients treated with dupilumab or methotrexate separately. All statistical calculations were performed with Stata statistical software (release 12.1; StataCorp, College Station, TX, USA).

### Ethics

This study was approved by the Regional Ethical Review Board at Karolinska Institutet, Stockholm. Informed written consent was provided by all participants.

## Results

In total, 12 patients (median age 50.5 years, range 23–60) had started treatment with dupilumab and eight patients (median age 56 years, range 43–81) had started treatment with methotrexate. Patients treated with dupilumab had been given at least one other systemic immunosuppressive treatment before dupilumab was considered: methotrexate (*n* = 12), ciclosporin (*n* = 6), omalizumab (*n* = 2), apremilast (*n* = 3), acitretin (*n* = 1), azathioprine (*n* = 1), and/or alitretinoin (*n* = 1). Half of the patients (*n* = 4) treated with methotrexate had not used any systemic immunosuppressive treatment at baseline. The other half had been treated with omalizumab (*n* = 1), apremilast (*n* = 1), acitretin (*n* = 1), and/or alitretinoin (*n* = 2). During the study period, none of the study participants used systemic glucocorticoids.

All patients were classified as having moderate or severe AD refractory to conventional treatment. Outcome measures for AD at baseline are shown in Table 1. Age, sex, EASI, POEM, VAS for pruritus, DLQI, and weight were comparable between patients with dupilumab and patients with methotrexate at baseline (start of treatment). DLQI was significantly higher among patients with methotrexate (*p* = 0.025), and EASI was significantly higher among patients with dupilumab (*p* = 0.045) (Table 1).

**Table 1:** Characteristics of patients with severe atopic dermatitis at start of treatment

	Methotrexate (*n* = 8)		Dupilumab (*n* = 12)		*p* value
	Mean	95% CI	Mean	95% CI	
Age (years)	56.4	46.0–66.7	46.9	39.6–54.2	0.189
Male sex (%)	62.5	24.5–91.5	83.3	51.6–97.9	0.304
EASI	15.3	8.9–21.7	27.6	18.7–36.5	0.045*
POEM	18.0	11.2–24.8	18.6	13.5–23.7	0.816
VAS	6.0	3.5–8.4	5.9	4.0–7.8	0.817
DLQI	17	12.3–21.7	9.4	4.5–14.3	0.025*
MADRS	11.7	6.3–17.1	12.8	3.8–21.9	0.421
Weight (kg)	75.3	64.7–85.8	84.8	73.4–96.1	0.142
BMI (kg/m^2^)	24.7	19.9–36.1	27.3	24.0–28.4	0.123

*Abbreviations*: *CI* Confidence interval, *EASI* Eczema area severity score, *POEM* Patient-oriented eczema measure, *VAS* Visual analogue scale, *DLQI* Dermatology life quality index, *MADRS* Montgomery-Åsberg depression rating scale, *BMI* Body Mass Index

Significant differences marked with *. *P* values calculated using two-sample Wilcoxon-Mann-Whitney rank-sum tests

**Fig. 1:**
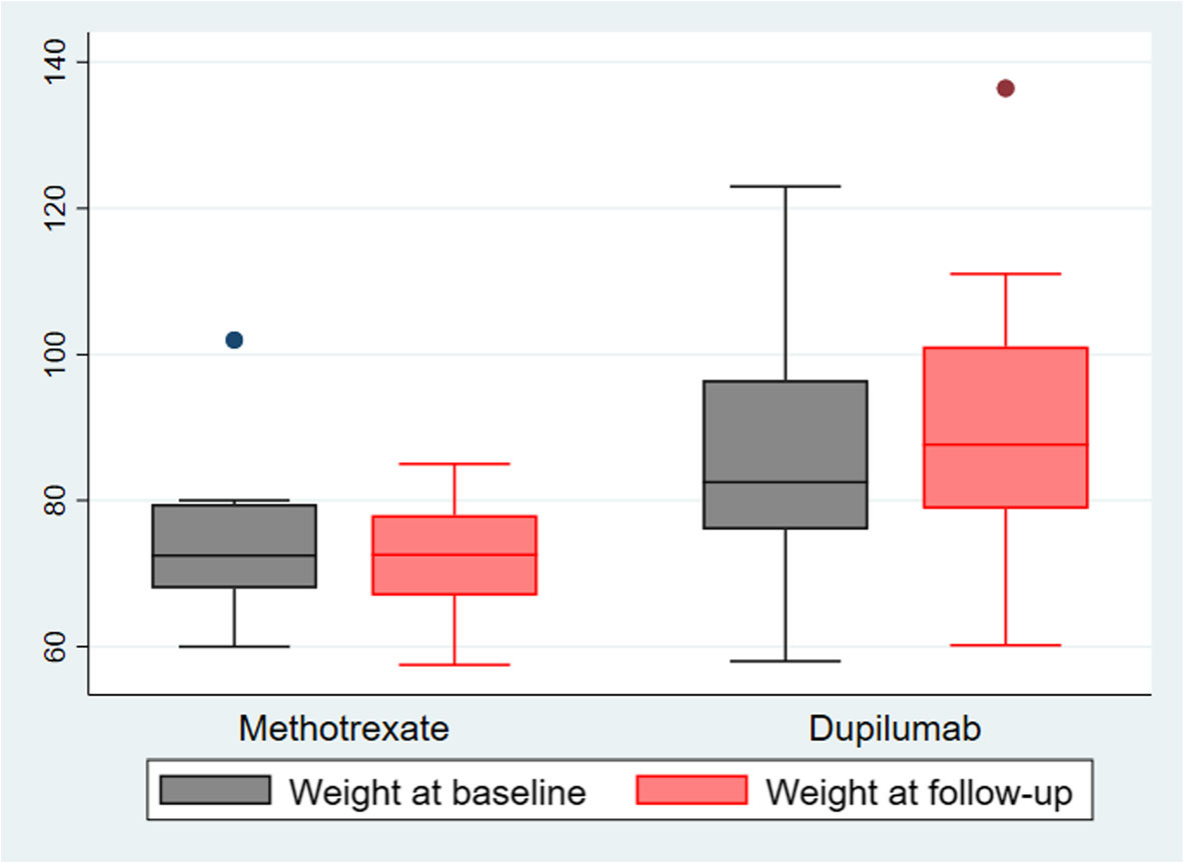
Median weight at baseline and follow-up 12 months later among patients with systemic treatment. The analysis is stratified by methotrexate (*n* = 8) and dupilumab (*n* = 12). Follow-up at 12 months (*n* = 15), at 9 months (*n* = 2), at 6 months (*n* = 3) due to missing value at 12 months or short time of follow-up. The difference in weight between baseline and follow-up was significant among patients treated with dupilumab (*p* = 0.002) but not among patients treated with methotrexate (*p* = 0.161). *P* values calculated using Wilcoxon matched-pairs sign-rank tests

**Fig. 2:**
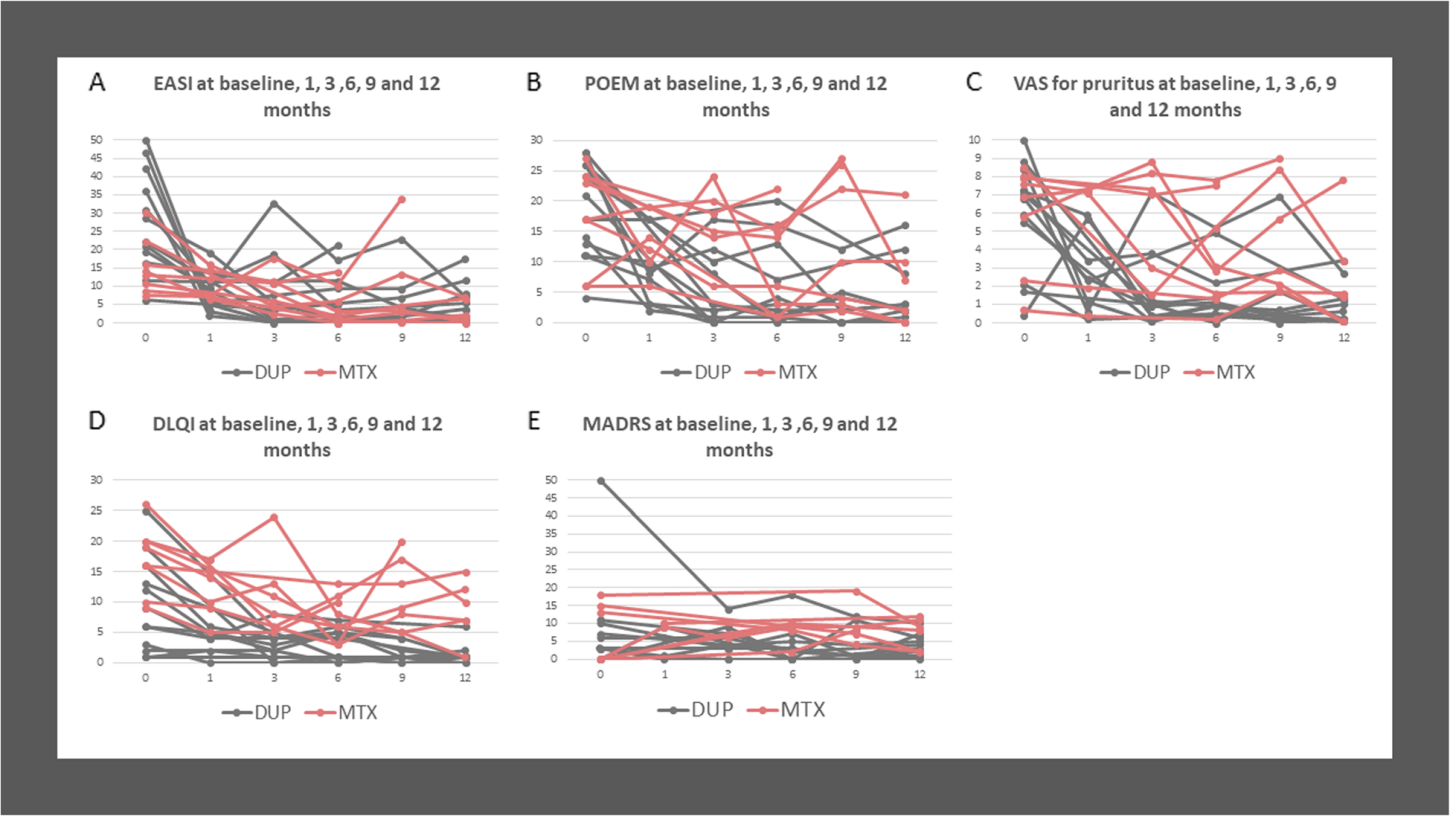
Outcome measures monitored during treatment with dupilumab or methotrexate in adults with atopic dermatitis. **a** Eczema Area Severity Index (EASI), **b** Patient-Oriented Eczema Measure (POEM), **c** Pruritus Visual Analogue Scale Score (VAS, 0–10 cm), **d** Dermatology Life Quality Index (DLQI), **e** Montgomery-Åsberg Depression Rating Scale (MADRS). Dupilumab (DUP) in gray (*n* = 12) and methotrexate (MTX) in red (*n* = 8)

At follow-up, patients treated with dupilumab had gained weight significantly (mean weight change: 6.1 kg, range [0.1–18.0], *p* = 0.002), while the weight of patients with methotrexate had not changed (mean weight change: − 3.1 kg, range [− 17.0–2.0], *p* = 0.161), Fig. 1. The majority of the patients showed a positive response to treatment (Fig. 2). Most patients treated with dupilumab had improved all outcome measures (EASI, POEM, VAS, DLQI, and MADRS) significantly. Moreover, at follow-up at 6, 9, or 12 months, they reached EASI-90 (*n* = 6), EASI-75 (*n* = 4), or EASI-50 (*n* = 1), and had a minimal clinically important difference in POEM (*n* = 10, 1 missing value). One patient did not respond to treatment with dupilumab. Patients treated with methotrexate reached EASI-90 (*n* = 3), EASI-75 (*n* = 2), or EASI-50 (*n* = 1) and had a minimal clinically important difference in POEM (*n* = 6). Two patients did not respond to treatment with methotrexate. Treatment was stopped and they switched to dupilumab instead.

### Weight change in relation to treatment response

We performed a sensitivity analysis among patients with AD successfully treated with dupilumab (*n* = 11) and methotrexate (*n* = 6), Table 2. Patients successfully treated with dupilumab were comparable with all patients treated with dupilumab (mean weight change: 6.6 kg, range [0.1–18.0]). Patients treated with methotrexate had a significant response to treatment (EASI, VAS, DLQI, and MADRS were significantly reduced) and tended to lose weight from baseline to follow-up (mean weight change: − 4.3 kg, range [− 17.0–2.0]), but not significantly.

**Table 2:** Outcome measurements among patients with atopic dermatitis successfully treated with methotrexate or dupilumab

	Methotrexate (*n* = 6)			Dupilumab (*n* = 11)		
	At start	At follow-up^a^	*p* value	At start	At follow-up^a^	*p* value
	Mean (range)	Mean (range)		Mean (range)	Mean (range)	
EASI^b^	16.8 (7.4–30.3)	2.8 (0–6.8)	0.028*	28.6 (6.2–50)	5.2 (0.3–17.5)	0.003*
POEM^c^	17.2 (6–27)	6.7 (0–21)	0.075	19.9 (11–28)	4.9 (0–16)	0.005*
VAS^c^	5.7 (0.7–8.5)	2.4 (0.1–7.8)	0.028*	6.3 (0.4–10)	1.1 (0.1–3.4)	0.007*
DLQI^b^	18.5 (10–26)	8.7 (1–15)	0.028*	10.2 (1–25)	1.6 (0–6)	0.005*
MADRS^c^	14.6 (13–18)	7.3 (3–12)	0.034*	13.7 (1–50)	3.9 (0–10)	0.028*
Weight^d^ (kg)	74.8 (60–102)	70.5 (57.7–85)	0.075	87.1 (58–123)	93.6 (65.5–136.4)	0.003*

*Abbreviations*: *EASI* Eczema area severity score, *POEM* Patient-oriented eczema measure, *VAS* Visual analogue scale, *DLQI* Dermatology life quality index, *MADRS* Montgomery-Åsberg depression rating scale

Significant differences marked with *. *P* values calculated using Wilcoxon matched-pairs sign-rank tests

^a^Follow-up after 12 months of treatment. Some patients had shorter follow-up and some variables were missing at 12 months, but collected at 6 or 9 months of follow-up

^b^Follow-up at 12 months (*n* = 16), at 9 months (*n* = 1)

^c^Follow-up at 12 months (*n* = 16), missing value at follow-up (*n* = 1)

^d^Follow-up at 12 months (*n* = 15), at 9 months (*n* = 1), at 6 months (*n* = 1)

### Appetite and sleep disturbance due to itching in relation to weight change

At baseline, the majority of patients treated successfully with dupilumab and methotrexate reported normal or increased appetite (7 of 11 and 3 of 5 [1 missing data], respectively). The other patients reported *slightly reduced appetite* (1 or 2 on scale 0–6). No patient reported *no appetite* or *need persuasion to eat* (≥3 on scale 0–6) (Additional files 1 and 2). In the dupilumab group, 3 of 4 patients still reported slightly reduced appetite at follow-up, and one patient reported normal or increased appetite. Among patients treated with methotrexate, 2 of 2 patients were improved and reported normal or increased appetite at follow-up. In the visual assessment, there was no correlation between reported appetite and weight change among patients treated with dupilumab or methotrexate.

Disturbed night sleep *Every day* last week (4, scale 0–4) was reported at baseline among 6 of 10 (1 missing data) patients treated successfully with dupilumab. At follow-up, one patient still reported disturbed night sleep *Every day* last week, and all others (*n* = 9) reported disturbed night sleep *No days* (0, scale 0–4). In all, 5 of 10 clearly improved their night sleep from baseline to follow-up. For five patients, night sleep was unchanged (one patient reported disturbed night sleep *Every day*, and the other four reported *No days*). Both patients with improved night sleep (*n* = 5) and those with unchanged night sleep (*n* = 5) gained weight (mean weight change: 7.2 kg and 3.8 kg, respectively) with no significant difference between the groups (*p* = 0.076). Among patients treated successfully with methotrexate, all reported disturbed night sleep in the last week at baseline (*1–2 days* [*n* = 1], *3–4 days* [*n* = 1], *5–6 days* [*n* = 2), *Every day* [*n* = 2]). All improved their night sleep from baseline to follow-up; two patients who reported *Every day* at baseline reported *1–2 days* and *3–4 days*, respectively, at follow-up. All others reported *No days* at follow-up. Patients treated successfully with methotrexate and who improved their night sleep during treatment did not gain weight (mean weight change: − 4.3 kg) (Additional files 1 and 2).

## Discussion

Patients with severe AD treated with dupilumab gained weight, a mean of 6.1 kg, over the course of 1 year on treatment. To the best of our knowledge, this is the first report on an association between treatment with dupilumab and weight gain. In this cohort study, with a limited number of participants, there was no obvious relation between weight gain and appetite, disturbed night sleep due to itching, or treatment response.

Our finding is in line with previous reports on weight gain during treatment with other biologics, such as anti-tumor necrosis factor (TNF)-α and IL-6 inhibitors. Patients with psoriasis increased their lean (fat-free) mass and fat mass during treatment with anti-TNF-α [6] and patients with RA treated with tocilizumab (an IL-6 inhibitor) for 1 year gained weight significantly, without change in fat mass. The RA patients gained mainly muscle mass and the authors suggested that inhibition of IL-6 could be effective in reversing muscle loss in RA patients [7]. In patients with IBD, weight gain as well as reverse growth failure in children are expected due to improvement of the symptoms during treatment. However, excessive weight gain has been reported in children with IBD exposed to anti-TNF-α [5]. In our study, the patients with AD treated with dupilumab seemed to put on more weight (mean weight gain: 6.1 kg) compared with RA patients treated with an IL-6 inhibitor (mean weight gain: 1.9 kg) [7], RA patients treated with anti-TNF-α (mean weight gain: 1.8 kg) [8], or patients with psoriasis treated with adalimumab (anti-TNF-α; mean weight gain: 2.2 kg) [23]. To our knowledge, there are no previous reports regarding an association between use of the anti-IL-4α receptor as a treatment target and weight change.

It is possible that disturbed night sleep due to itching and continuous inflammation increase energy consumption, meaning that the energy need is reduced upon improvement. Thus, if patients on successful treatment do not change their diet, they will gain weight. Patients with AD scratch their skin during night sleep more often than healthy controls (24 min vs. 2 min, respectively) [10] and one small study (nine patients with AD) showed an increased oxygen consumption due to scratching during night sleep [11]. However, Hon et al. compared 13 AD patients with eight healthy controls and did not find an increased resting energy consumption, increased oxygen consumption, or increased carbon dioxide production during sleep among AD patients. They speculated that altered energy expenditure during night sleep is unlikely among children with AD [24]. In our study, patients with severe AD successfully treated with dupilumab gained weight, but patients successfully treated with methotrexate did not. The weight gain among patients with dupilumab was not significant associated with improvement in night sleep. Therefore, it is unlikely that reduction in energy expenditure explains the findings in our study. The larger weight gain as compared with that among RA patients and psoriasis patients treated with other biologics supports the idea that weight gain could be a direct side effect of dupilumab. Inhibition of the activation in brown fat is a possible mechanism behind the weight gain [13]. However, this area needs further exploration.

The strengths of this study were that data on weight and outcome measures were registered consecutively for all patients with AD treated with systemic drugs in a clinical setting, and that we could use patients who received other treatments for comparison. The outcome measures for AD used in this study are all validated [18, 19, 25, 26], and EASI and POEM are recommended by the group Harmonizing Outcome Measures for Eczema [18], which is also a strength. The small number of participants is a limitation, especially the few patients successfully treated with methotrexate used for comparison. Another limitation is that patients with dupilumab treatment differed from patients with methotrexate regarding EASI and DLQI at baseline, and tended to differ in weight, although not significantly. However, the treatment response was significant and comparable among patients who responded to treatment. Nausea is a common adverse event among patients using methotrexate [27]. Among patients treated with methotrexate, it is therefore possible that effects or side effects of the treatment biased the relation between weight gain and appetite or energy consumption. Thus, we cannot rule out that the difference in weight gain between the groups might be explained by methotrexate rather than by dupilumab. However, this is less likely since the patients, regardless of treatment, reported similar appetite levels. Furthermore, it is our experience that patients suffering from severe nausea will switch drugs before 1 year of treatment has passed.

## Conclusion

We report weight gain as an unexpected and unexplained side effect of dupilumab treatment, from a clinical setting among patients with severe AD. Therefore, we recommend monitoring of weight during treatment with dupilumab and informing patients of this possible side effect. To our knowledge, this is the first report on a possible association between weight gain and treatment with dupilumab and the extent of the association is yet to be seen, as is the mechanism behind this finding. Thus, larger studies are warranted to confirm this finding.

## Supplementary information

**Additional file 1. **Change in weight, appetite, and sleep disturbance from baseline to follow-up 12 months later among patients with atopic dermatitis successfully treated with dupilumab (*n* = 11).

**Additional file 2. **Change in weight, appetite, and sleep disturbance from baseline to follow-up 12 months later among patients with atopic dermatitis successfully treated with methotrexate (*n* = 6).

## Data Availability

The datasets used and/or analyzed during the current study are available from the corresponding author on reasonable request.

## Abbreviations

AD: Atopic dermatitis
BMI: Body Mass Index
CI: Confidence interval
DLQI: Dermatology life quality index
EASI: Eczema area severity score
IBD: Inflammatory bowel disease
IL: Interleukin
MADRS: Montgomery-Åsberg depression rating scale
POEM: Patient-oriented eczema measure
RA: Rheumatoid arthritis
TNF: Tumor necrosis factor
VAS: Visual analogue scale
