# Supplementary material for: Weight gain in patients with severe atopic dermatitis treated with dupilumab: a cohort study
Source: BMC Dermatol. 2020 Sep 22;20:8. doi: 10.1186/s12895-020-00103-0 (PMC7510313; doi:10.1186/s12895-020-00103-0)
Supplement: Supplementary file 1 — Additional file 1. Change in weight, appetite, and sleep disturbance from baseline to follow-up 12 months later among patients with atopic dermatitis successfully treated with dupilumab (n = 11). [file 12895_2020_103_MOESM1_ESM.docx]

**Additional file 1** Change in weight, appetite, and sleep disturbance from baseline to follow-up 12 months later among patients with atopic dermatitis successfully treated with dupilumab (n=11).

|  | Weight (kg) | | |  | Appetite^a^ (scale 0–6) | | |  | Disturbed night sleep^b^ (scale 0–4) | | |
| --- | --- | --- | --- | --- | --- | --- | --- | --- | --- | --- | --- |
| Patient | Baseline | Follow-up | Difference |  | Baseline | Follow-up | Difference |  | Baseline | Follow-up | Difference |
| 1 | 123 | 136.4 | 13.4 |  | 2 | 0 | -2 |  | 4 | 0 | -4 |
| 2 | 97 | 102.2 | 5.2 |  | 1 | 2 | 1 |  | 0 | 0 | 0 |
| 3 | 82 | 100 | 18 |  | 2 | 1 | -1 |  | 4 | 0 | -4 |
| 4 | 96 | 98.0 | 2.0 |  | 0 | 0 | 0 |  | 0 | 0 | 0 |
| 5 | 82 | 88.1 | 6.1 |  | 0 | 0 | 0 |  | 4 | 0 | -4 |
| 6 | 85 | 87.2 | 2.2 |  | 0 | 0 | 0 |  | 4 | 4 | 0 |
| 7 | 83 | 84 | 1 |  | 0 | 0 | 0 |  | 4 | 0 | -4 |
| 8 | 78 | 78.1 | 0.1 |  | 0 | 0 | 0 |  | 0 | 0 | 0 |
| 9 | 58 | 65 | 7.5 |  | 0 | 1 | 1 |  | 4 | 0 | -4 |
| 10 | 100 | 111 | 11 |  | 0 | - | - |  | 3 | - | - |
| 11 | 74 | 79.6 | 5.6 |  | 1 | 1 | 0 |  | 0 | 0 | 0 |

^a^Appetite based on response to MADRS questionnaire. “*Reduced appetite compared with when feeling well? Scale 0–6; 0: Normal or increased appetite; 2: Slightly reduced appetite; 4: No appetite / food is tasteless 6: Need persuasion to eat.”*

^b^Sleep disturbance based on response to POEM questionnaire: *“Over the last week have many nights has your sleep been disturbed because of the eczema? Scale 0–4; 0: No days 1: 1–2 days 2: 3–4 days 3: 5–6 days 4: Every day”*
